# Fabricating Natural Polymeric Encapsules for Pest Control Uploaded with 1,8-Cineole Extracted from Eucalypt Ecotypes’ Leaves Using Innovative Microwave Tool

**DOI:** 10.3390/polym17091182

**Published:** 2025-04-26

**Authors:** Sherif S. Hindi

**Affiliations:** Department of Agriculture, Faculty of Environmental Sciences, King Abdulaziz University (KAU), P.O. Box 80208, Jeddah 21589, Saudi Arabia; shindi@kau.edu.sa; Tel.: +966-56-676-0086

**Keywords:** eucalyptus ecotypes, microwave extraction, essential oil, 1,8-cineole, microcapsules, alginate, guar gum, bioassay screening

## Abstract

This research explores the potential of green encapsules uploaded with eucalypt essential oil (EEOs) in enhancing their functionality and application in pest control, focusing on suitable ecotype selection from King Abdulaziz University (KAU) campus, Hada Al-Sham (HAS) village, and Briman district as well as optimizing extraction processes. Eucalypt hybrids’ leaves were collected from three different sites, and the EEOs were extracted using microwave-assisted steam distillation (MASD) and electric steam distillation (ESD) techniques. The physical and chemical properties of the EEO were determined. The identification of volatile chemical ingredients in the resulting EEOs was conducted using GC/MS after saponification and methylation procedures, and the ingredients were compared to those obtained from *Eucalyptus globulus* Labill, the ideal species containing the 1,8-cineol, the principal compound in its essential oil. The 1,8-cineole was found to be the major chemical constituent of the EEOs all over the two extraction methods, regardless of the ecotypes examined, and was interfered with other minor components such as 3-carene, α-pinene, α-myrcene, D-limonene, and α-terpinene. Eucalypt ecotypes grown at Hada Al-Sham village had the highest cineole content (59.29%) among the other sites studied. Compared to the ESD technique, MASD showed much promise because it is simple, facile, more ecofriendly and cost-effective, it kept oils true to their original form, and it allows to warm larger machines and spaces. The polymeric encapsules of either guar gum crosslinked by borax or sodium alginate crosslinked by calcium chloride were fabricated. Moreover, a bioassay screening of the encapsules uploaded with 1,8-cineole was evaluated against termite infection. The encapsules were found to be versatile tools with a wide range of applications; in particular, the alginate encapsules displayed superior characteristics. Furthermore, regardless of the encapsule type and the exposure duration, the mortality (%) of the insects was exceeded significantly for the high cineol concentrations compared to the lower ones for both alginate-based encapsules (ABEs) and guar gum-based encapsules (GGBEs). The higher the cineol concentrations, the higher the mortality percent of the termites. This finding can be attributed to the rapid toxic effect of the cineol compound at higher concentrations.

## 1. Introduction

The Eucalyptus genus offers significant ecological, economic, and social benefits. Its diverse applications range from timber and paper products to essential oils and medicinal uses, demonstrating its versatility and importance. However, it is also essential to manage Eucalyptus plantations responsibly, as their introduction to non-native environments can sometimes lead to ecological imbalances. Properly managed Eucalyptus cultivation can contribute significantly to sustainable development and environmental protection efforts [1,2]. The choice of eucalypt ecotype can have a profound impact on the quality and quantity of essential oil obtained, as well as its suitability for different applications. In addition, variability in both composition and production of essential oils can be attributed to intrinsic factors such as genetics, subspecies, and the age of the plant, as well as extrinsic factors like climate, cultivation conditions (including geographical origin), and the methods employed for isolation [1,2]. The EEOs are produced as a result of the secondary metabolic activities of their leaves [3]. Through the transpiration process, leaves release water vapor into the air that helps to regulate the plant’s temperature and contributes to the water cycle and volatile compounds, which includes but is not limited to terpenoids, aldehydes, alcohols, esters, phenolic compounds, nitrogen-containing compounds, as well as green leaf volatiles that are released upon leaf damage. Moreover, Eucalyptus leaves contain specialized glands known as secretory cavities or oil glands responsible for producing the essential oils that are the characteristic aroma of Eucalyptus species. In addition to these oil glands, Eucalyptus leaves may also contain resin ducts, which help in the production and storage of resin, further contributing to the plant’s defensive qualities. The combination of oil and resin contributes to the overall healing properties and aromatic qualities of Eucalyptus [1,2,3].

In addition, *Eucalyptus globulus*’ essential oil is characterized by its prominent component, 1,8-cineole (eucalyptol), a monoterpene found in the essential oils of various plants, which was determined to be 60–85 % for copious trees as indicated by several researchers [4,5,6,7,8].

From a chemical composition perspective, the volatile oil of *Eucalyptus citriodora* has been documented to predominantly contain citronellal, and its concentration has been observed to vary seasonally [9,10]. The fluctuating levels of this compound serve as valuable indicators for accurately distinguishing *E. citriodora* essential oil from that of other Eucalyptus species.

Concerning usage and applications of the EEOs, they have been used for cosmetics [11], aromatherapy [9], pesticide industries [9], and for honeybee pastures. Furthermore, they have potential utilizations for anesthetics [2], antiseptics, and astringents [10,12]. They are popular in the food industry for antioxidant and antibacterial properties [8], as well as their pleasant flavors [13] in food industry. Moreover, they can be used as antifungal applications [14] as well as in pharmaceutical products, with extensive research exploring their biotic actions and components [15,16,17]. Their utilization as bio-insecticides [18] as well as the antibacterial efficacy and of the preparations, as well as individual/pure essential oils, can be influenced by the presence and concentration of α-pinene [2,19,20].

Essential oil extraction is a complex process involving classical and innovative techniques. Classical methods like hydro-distillation and steam distillation rely on heat and water while green methods like ultrasound-assisted and microwave-assisted extraction use energy-efficient sources. Supercritical fluid and subcritical liquid extraction allow non-polar components to be extracted. Hydro-distillation is the most commonly used method due to its accessibility and cost-effectiveness. Eucalyptus’ essential oil extraction involves crushing, drying, and distillation. Traditional methods like maceration, oil infusion, and steam distillation are labor-intensive and expensive. Microwave-assisted steam distillation (MASD) is a fast and efficient alternative to traditional methods, combining steam distillation with microwave heating, reducing extraction time, enhancing selectivity, and increasing essential oil yields. It has gained attention as an efficient and environmentally friendly alternative for extracting essential oils from botanical sources. In a microwave reactor, electromagnetic energy is transformed into heat, causing the sample to rupture and release essential oil [21,22,23,24,25].

The MASD has several key points regarding the novelty and advantages of microwave oil extraction such as improving efficiency, reducing the extraction period, as well as solvent usage, energy savings, enhancing the quality of extracts, scalability, simplicity and process integration, modifiability, and innovation in byproduct utilization [26,27,28,29,30,31,32,33,34,35,36,37]. Furthermore, it has other benefits such as its accessibility, sustainability, cost-effectiveness, and labor-intensiveness; it also shortens the extraction time, enhances selectivity, increases essential oil yields, is environmentally friendly, and does not harm the quality or chemical composition of the oils [22,23].

This technique involves placing samples in a microwave reactor without solvent, converting electromagnetic radiation into heat within a frequency range of 300 MHz to 300 GHz, increasing cell temperature, and releasing essential oils. This method has been successful in extracting essential oils from various plant materials, including orange, laurel, lemon, mint, rosemary, and basil [26,27,28,29,30,31,32,33,34,35], reducing extraction duration, and improving specificity [36,37].

Microcapsules have numerous applications in daily life, including drug delivery, cell therapy, food industry, biotechnology, cosmetics, and wastewater treatment. They allow the controlled release of pharmaceuticals, protect against degradation, immobilize enzymes for biocatalysis, and remove pollutants or heavy metals in cosmetic products [38,39,40]. It is a technological process centered around the enveloping of solid, liquid, or gaseous particles with an encapsulating agent, serving as a protective barrier that entirely shields the core material from the surrounding external environment. It is widely used in the food and pharmaceutical industries to protect and deliver bioactive compounds, including essential oils, in a controlled and targeted manner [41,42]. Gum Arabic, agar, alginate, proteins, carbohydrates, lipids, and dextrin are among the materials employed as encapsulating agents in the microencapsulation process [43,44,45,46,47]. The physicochemical properties of microcapsules are determined by the encapsulating and active agents, with wall material forming a cohesive film. Microencapsulation allows for the regulated, precise, and controlled discharge of active ingredients, triggered by temperature fluctuations, solubility changes, pH levels, or wall material biodegradation, and can occur at specific times or under specific conditions [47].

Microencapsulation methods can be divided into chemical, physicochemical, and mechanical methods that differ in their speed, ease of use, reproducibility, and scalability for industrial applications such as spray drying and coacervation [48]. Microencapsulation emerges as a viable solution to address numerous challenges associated with the utilization of essential oils. The use of essential oils is considerably impeded by their pronounced volatility and chemically unstable attributes [49,50,51].

Sodium alginate is a natural polysaccharide derived from brown algae. It is the main component forming the microcapsule shell. Moreover, it is a linear polymer composed of mannuronic acid and guluronic acid residues. Calcium chloride (CaCl_2_) is the source of calcium ions (Ca^2+^) essential for crosslinking the alginate molecules. Alginate-based encapsules have a number of benefits, including biocompatibility, which describes how well the body tolerates them in general, ease of formation, which is straightforward and affordable, gelation that takes place in mild conditions (neutral pH, room temperature), which makes them appropriate for encapsulating sensitive substances, and tunable 96 properties, which can be customized by varying the process parameters [52].

Regarding the bioassay screening of cineole against termites, it has been examined for its efficacy as a natural insecticide. Bioassay screening for cineole’s effectiveness against termites can contribute to developing environmentally friendly pest control strategies [53,54,55].

A mortality assessment was performed to study factors affecting termite mortality and behavior such as mortality rates, cineole’s dose–response, termites’ exposure time. Data collection can be conducted by monitoring and recording termite mortality after treatment at specified intervals as well as observing behavioral responses for repellency, such as changes in movement, feeding behavior, or the avoidance of treated areas [53,54,55].

The objectives of this study were to identify the Eucalyptus species with the highest cineol concentration, investigating the potential of microwave-based heating for constructing cost-effective, large-scale machinery, thus facilitating the efficient mass production of essential oils and exploring potential applications of the microencapsulated Eucalyptus essential oil in guar gum or calcium alginate for efficacy and stability in pest control applications.

## 2. Materials and Methods

### 2.1. The Management Plan

**Figure A1:**
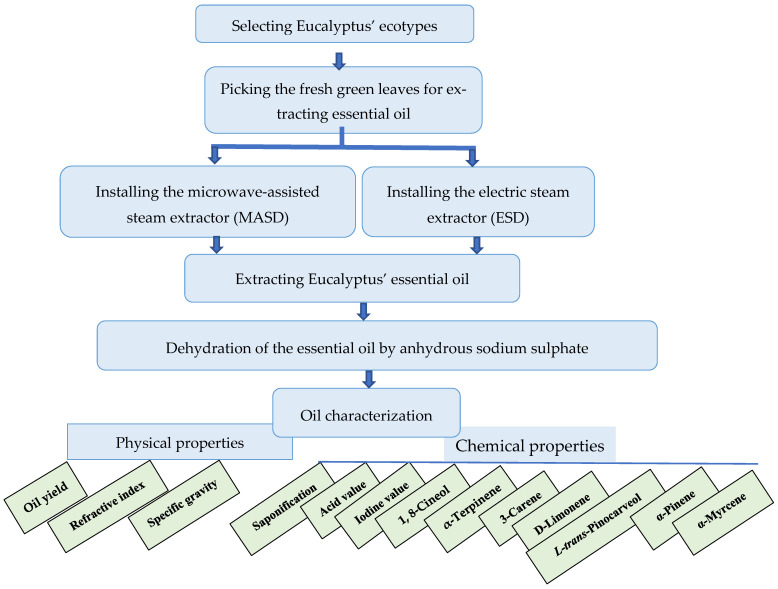
The management strategy outlines the techniques for synthesis and evaluation of the Eucalyptus’ essential oil investigating the effectiveness of two extraction methods: microwave-assisted steam distillation and electric steam distillation.

The management strategy for synthesis and evaluation of the Eucalyptus’ essential oil (EO) using the two extraction methods, namely, microwave-assisted steam distillation (MASD) and electric steam distillation (ESD), is outlined in Figure A1. In addition, comparisons between extraction methods of essential oils were shown in Table S1.

The electric components of the microwave generator unit (MGU) used for heating the extraction vessel of the MASD are presented in Figure S1. Moreover, the working theory of the novel microwave irradiation apparatus in heating botanic tissues and enhancing extracting EEOs by adjusting the hot and cold spots that arose within the botanic tissues are shown in Figure A3.

Furthermore, the protocol of preparing methyl esters of the essential oils (EOs) for accurate analysis by GC-MS is shown in Figure S2.

In addition, split-plot statistical designs for studying the obtained natural products, namely, EOs and polymeric encapsules, are presented in Tables S2 and S3, respectively.

**Figure A2:**
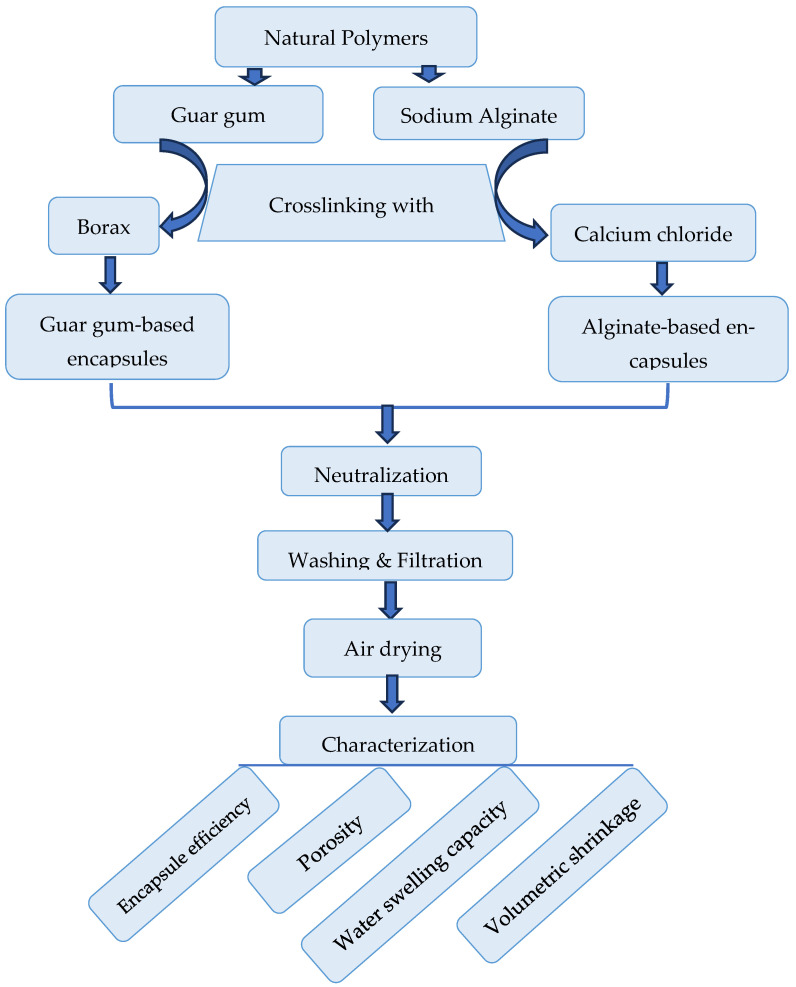
The management strategy outlines the techniques for investigating the polymeric encapsules fabricated from each of the guar gum and alginate and uploaded with 1,8-cineol.

The management strategy illustrating the techniques for investigating the polymeric encapsules fabricated from each of the polymeric encapsules used (guar gum and alginate) uploaded with 1,8-cineol is presented in Figure A2.

### 2.2. Tree Species

**Figure 1:**
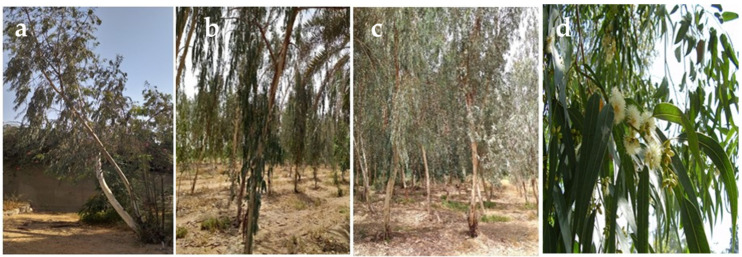
The eucalypt ecotypes grown at (**a**) KAU campus, (**b**,**c**) Hada Al-Sham (HAS), and (**d**) Briman, used for extracting essential oil from their leaves.

*Eucalyptus* ecotypes were selected (Figure 1), identified botanically, and were specified for the present investigation.

The trees were selected from three sites in the western region of Saudi Arabia. The first site chosen was King Abdullaziz University (KAU) campus (Figure 1a). The second site investigated was the Agricultural Research Station (ARS) that belongs to the KAU at Hada Al-Sham village (Figure 1b,c) in Al-Jomoom Governate, about 120 km away from Jeddah (at a latitude of 21° 46′.839 N and a longitude of 39° 39′.911 E above the sea level by 206 m). Furthermore, the recreation forest at Briman district (Figure 1d) was the third place considered. In addition, *Eucalyptus globulus* Labill trees were chosen from those grown at the ARS.

#### 2.2.1. Sprouts of the Selected Trees

Three sprouts (two years old) were chosen from each tree. The diameter outside bark of the selected trees ranged from 8 to 10 cm. Each of the selected sprouts was cut at height of 10 cm above its base connection with the main trunk. The height between the sprout base and ground level ranged from 30 to 40 cm.

#### 2.2.2. Leaves’ Raw Materials

The Eucalyptus’ leaves were collected randomly in April 2023 and used to extract essential oils. Three trees were selected from each of the three locations. The ages of the selected trees were about 15 years except for those grown at the KAU campus whereby their age was about 20 years.

The fresh leaf samples were botanically identified [56], weighed, and cleaned to exclude any extraneous substances. The sequential steps included the preparation and extraction process of essential oil from Eucalyptus leaves. After that, the collected leaves were air-dried, and the resultant essential oil were collected, purified, and characterized.

### 2.3. Essential Oil Extraction Process and Apparatus

Several techniques available traditionally are presented in Table S1. In the current study, two methods were used for heating the extraction tank (the autoclave vessel), namely, electric steam distillation (ESD) and microwave-assisted steam distillation (MASD) utilizing the same steam distillation apparatus although they diverge in terms of the heating instrument required to heat the extruder’s colander inside the machine.

**Figure 2:**
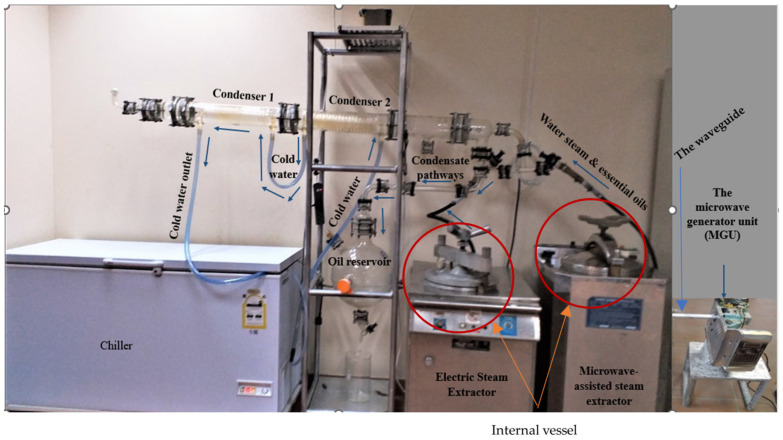
Electric- and microwave-assisted steam distillation apparatus used for distilling the essential oil from Eucalyptus leaves showing the internal vessel of the steam–water distiller.

The fresh leaves (2 kg) were distilled in a steam–water distiller with an internal vessel of 72 L in volume using about 10 L of distilled water (Figure 2) for 3 h. The collected essential oil was dehydrated over anhydrous sodium sulfate and stored at 4 °C before analysis [57].

The equipment used for the extraction of Eucalyptus’ EO consists of an autoclave apparatus, heated with a microwave device or an electric coil, a Clevenger distillation apparatus, and a flask (oil reservoir), as shown in Figure 2.

**Figure A3:**
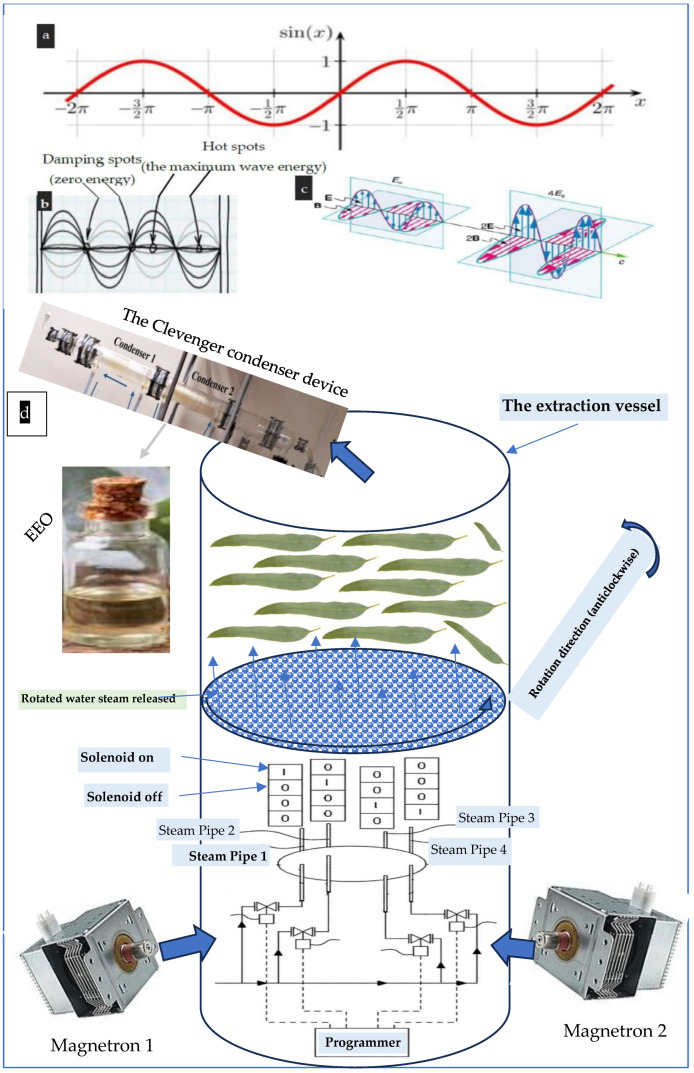
The microwave beam: (**a**) sinusoidal wave curve (SWC), (**b**) energy level along with the microwave sinusoidal curve (SWC), (**c**) the proportionality between square amplitude and the energy carried by the wave, and (**d**) the innovative gaseous agitation device used to rotate the evaporated water steam-bearing oils.

The microwave-assisted steam distillation (MASD) was designed to rely on a microwave generating unit that emits microwave beams to achieve the desired temperature of the extraction process, as shown in Figure 2, Figure A3, and Figure S1. The installation of MASD was carried out, followed by omitting the electric heater, and the autoclave was allowed to be heated by a microwave beam directed towards using an ideal waveguide. Concerning electric steam distillation (ESD) apparatus, the ESD (Figure 2) was also used for distilling the essential oil from Eucalyptus’ leaves. It uses an indirect electric current heater for this function.

Subsequently, a water-distillation process was conducted using a Clevenger extractor device that is used to condense and separate the oil and aqueous phases, which occur externally to the heating tool, resulting in the extraction of greenish-yellow oil. The extraction process was conducted for a duration of three hours, and the resulting essential oil was subjected to dehydration using anhydrous sodium sulfate. Subsequently, the oil was kept at a temperature of 4 °C prior to analysis [57]. Following a one-hour settling period, the oily supernatants were separately collected and subjected to filtration using vacuum filters to eliminate any impurities present in the oil [57,58,59,60].

The microwave generator unit (MGU) is tasked with the conversion of alternating electric current (AC) into microwave radiation. It consists of five components, namely, magnetron, transformer, capacitor, diode, and waveguide. The unit shown in Figure S1a comprises the below-mentioned components. The high voltage transformer, identified as 1000E–1E, is intended for use with a power supply of 220 V and a frequency of 60 Hz (Figure S1b). Moreover, the magnetron 2M214 39F(06B), specifically coded as 2B71732E, is designed for use in LG microwave ovens. It operates at a power of 900 W, with an anode voltage of 4.20 kVp and a frequency of 2460 MHz (Figure S1c). The high voltage capacitor is rated for an alternating current (AC) voltage of 2100 V, with a capacitance of 1 µF ± 3%. It is designed to operate at a frequency of 50/60 Hz and has a resistance of 10 MΩ (Figure S1d). Furthermore, in this experimental setup, a compact metallic waveguide was used to facilitate the transmission of microwave power from the magnetron to the extraction vessel containing aromatic tissues in the extraction apparatus [61,62].

Following a settling period of one hour, the oily supernatants were separately collected and subjected to filtration using vacuum filters to eliminate impurities presented in the oil. After the crude oil was received, it underwent weighing and subsequent storage prior to undergoing various characterizations. However, several documented methodologies employed by researchers operating within similar and interconnected disciplines are shown in Table S1.

### 2.4. Characterizations of the EEOs

Evaluation of the EOs’ effectiveness by determining several physical, chemical, anatomical, and spectroscopic properties is presented in Figure A1.

Determination of oil specifications was determined according to the procedures provided by other researchers [62,63,64,65,66,67,68,69].

In addition, the ASTM standard methods were applied for specific gravity (SG), saponification value (SV), the acid value (AV), and iodine number (IN) according to ASTMs [70,71,72,73,74].

#### 2.4.1. Physical Characterization of the EEOs

Concerning the yield of the essential oil (YEO), it was calculated using the following formula [56]:

YEO, % = (W_1_/W_2_) × 100,

where W_1_ is the essential oil weight (g) and W_2_ is the fresh leaf weight (g).

Moreover, relating to the specific gravity (SG) of the EO, a known weight-glass tube was filled first with essential oil and weighed (W_1_). Subsequently, the same tube glass was filled up to the same volume of deionized water and weighed (W_2_). Then, the specific gravity (δ) was calculated using the following equation [63,64,65]:

SG = (W_1_/W_2_),

where W_1_ is the weight of a certain volume of the essential oil (g), and W_2_ is the weight of the same volume of deionized water.

Furthermore, considering the refractive index (RI), a refractometer model No. 922313 (Bellingham and Stanley Ltd., London, UK) was used for the determination of the Eucalyptus essential oils’ RI at 40 °C [64,65].

#### 2.4.2. Chemical Analysis of the EEOs

Qualification of the obtained EO was analyzed by GC-MS after preparing their methyl esters as illustrated in Figure S2 according to the characterizations performed by Hindi et al. [62].

The saponification value (SV) was measured and determined using the following mathematical expression:

SV, mg KOH/g oil = [56.1N × (V_1_ – V_2_)/W],

where V_1_: The solution volume used for the blank test. V_2_: The solution volume used for fixed oil. N: The actual normality of the HCl used. W: The fixed oil weight.

The determination of the acid value (AV) of EEO was determined using the next equation [62,75]:

AV, mg KOH/g oil = [5.61(V × N)/W],

where V: Volume of KOH IN mL. N: normality of KOH. W: the fixed oil weight.

The iodine number (IN) is a quantitative measure of the degree of unsaturation, specifically the presence of double bonds, in a given essential oil. Oils characterized by a higher IN exhibit a greater abundance of double bonds [74]. It was determined by the quantity of iodine that undergoes reaction with 100 g of the oil. The IN was determined using the procedure previously outlined [59,62,74,76] as follows:

IN, g I_2_/100 g oil = [12.69 × C × (V_1_ – V_2_)/W],

where C, V_1_, and V_2_ are the parameters of sodium thiosulphate, C is the concentration, V_1_ is the volume used for the blank test, V_2_ is the volume used for the fixed oil, and W is the fixed oil weight.

##### Fractionated Compounds of the EEOs by GC-MS Analysis

The EEO was analyzed on a Shimadzu GC-17A gas chromatograph, SpectraLab Scientific Inc., (Markham, ON, Canada) coupled to a mass spectrometer operated in negative chemical ionization mode. A fused silica capillarity column with chemically bonded phases was used for the EEO’s constituent separation. After injecting the EEO through autosampler, it was analyzed with HP5 MS column. The yields of the chemical constituents of the volatile oils were determined using the peak area normalization method [65,66,67,68,69] as illustrated in Table 1. Furthermore, preparation of methyl esters of the essential oils was performed for facilitating their analysis by using GC-MS (Figure S2).

Mass spectra were compared with those from the National Institute of Standards and Technology (NIST), USA, and retention indices were compared with data from the scientific literature to determine the composition of the sample [67,68]. The peak area normalization technique was used to calculate the yields of the chemical components present in the volatile oils [66,67,68,69,77].

#### 2.4.3. Anatomical Features of the Leave-Tissues Bearing the EEOs

##### Optical Microscopy

The preparation of anatomical samples for microscopy involved three processes: prefiltration, infiltration, and polymerization as indicated by Merela et al. [78] with some self-modifications. The samples were dehydrated in 70% ethanol, then in a 1:1 propylene oxide-resin blend for 30–40 min, and finally embedded into a pure resin block. Concerning sectioning and slide preparation, Lecia HistoCore Nanocut microtome (Leica Biosystems Deer Park, IL, USA), was used to make an ultrafine section (~10 µm) of a leaf tissue using a diamond cutting knife with an angle of 3–8°. Then, slices were collected and stained using toluidine blue. Once a golden ring appeared on the outside of pigment droplet, the staining was completed. The optical speculation system consisted of a light microscope (CE–MC200A) with suitable vision system (OPTIKA PRO 5 Digital Camera–4083.12) using a Vision PRO 4 software.

###### Scanning Electron Microscopy (SEM)

The surface appearance and anatomical features of biopolymeric structured leaf tissues were examined using SEM imaging technology, and the ensuing image analysis proved to be highly successful [4].

A compact portion of a leaf, about 3 mm in length, was isolated, air-dried before embedding and fixation, attached to a double-sided carbon tape, and sputtered by gold to enhance its electric conductivity. Subsequently, the sample to be examined by the SEM was placed on an aluminum stub analysis. A Quanta FEG 450 scanning electron microscope (SEM) type, produced by FEI, based in Amsterdam, Netherlands, was used to examine the materials. The accelerating voltage used to test the microscope ranged from 5 to 20 kV.

### 2.5. Microencapsulation

**Figure 3:**
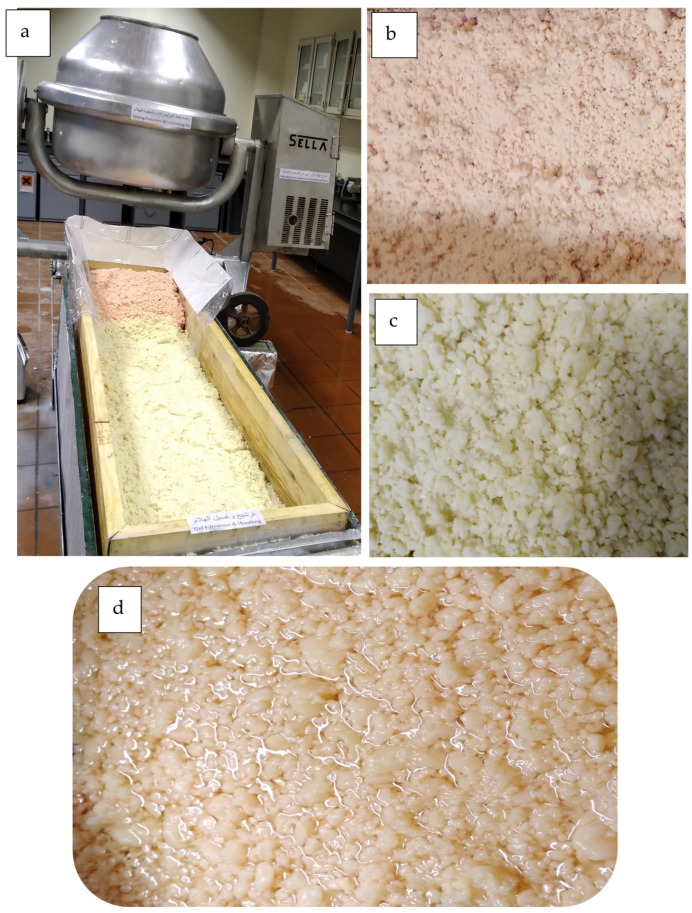
Microcapsules’ fabrication process: (**a**) the mechanical mixer used to blend polymeric matrix, (**b**) the air-dried alginate-based encapsules (ABEs), (**c**) the air-dried guar gum-based encapsules (GGBEs), and (**d**) immersing ABE in essential oil solution.

The management plan for fabricating and investigating the microcapsules fabricated from each of alginate-based hydrogels (ABHs) and guar gum-based hydrogels (GGBHs) can be seen from Figure 3 and Figure A2 according to Wibowo et al. [79] with some modifications.

Each of the two microcapsules’ systems of alginate-based hydrogel as well as guar gum-based hydrogel was synthesized by crosslinking with calcium chloride and borax, respectively, due to their known biocompatibility, ease of formation, and ability to encapsulate Eucalyptus’ essential oil [52,79]. Accordingly, the following compounds were used: (a) commercial guar gum (Foods Alive, Angola, IN, USA), (b) borax (di-sodium tetraborate decahydrate, 99.0%, purity), (c) sodium alginate (Cavex, Haarlem, The Netherlands), and (d) calcium chloride, 94% (Al Rakah Al Shamiyah Dist., Dammam, Saudi Arabia). The chemical information of the two polymers and their crosslinkers used in fabricating the encapsules’ hydrogels can be seen in Table A1.

First, diluted cineol solution (5%, vol/vol) was prepared, and a suitable quantity was used to be incorporated within the encapsules’ skeletons before crosslinking of alginate or guar gum. Sodium alginate or guar gum powders were dispersed in adequate volume of the prepared cineol solution, and concentrations of each alginate and guar gum powders were adjusted up to 5% (wt/wt), depending on desired microcapsule properties such as size, shell thickness, capsules’ porosity, and mechanical strength.

After synthesizing good emulsions of each alginate or guar gum, each polymeric solution was permitted to be crosslinked using calcium chloride solution (5%, wt/wt) for alginate as well as borax termed as sodium tetraborate (5%, wt/wt) in which the [B(OH)_4_]^−^ anion was reacted with the guar gum molecule.

It is worth mentioning that dispersing a fine powder like alginate or guar gum in water, to obtain homogeneous solution, was achieved by using homogenizer. Moreover, to prevent producing solid aggregates within alginate or guar gum solution intended to be crosslinked, an innovative mixing manner of powder with water was conducted. First, a known volume of deionized water was centrifuged by the homogenizer, which was adjusted to make a central vortex in the water. Then, the desired amount of a powder was added slowly, producing a homogeneous solution that gave the best encapsule quality when crosslinked. Finally, the microcapsules were then air-dried and then freeze-dried until its moisture content reached about 5% [79].

#### 2.5.1. Characterization of the Microcapsules

The encapsulation efficiency (EE) percentage property was calculated by dividing the volume of the essential oil in the encapsule after a known period over its initial volume within the same encapsule [79].

EE, % = [(W_1_ – W_2_)/W_2_] × 100

W_1_: Volume of the essential oil in the encapsule after a known period, cm^3^. W_2_: Volume of the initial volume of essential oil within the same encapsule, cm^3^.

Concerning porosity percentage of encapsules, this was determined using mercury displacement using Amsler volume meter.

VVE, % = (1 – V_od_) × 100

V_od_: Oven-dried volume of the encapsules.

Water swelling capacity (WSC) of an encapsule refers to its ability to absorb water and swell in volume as a result. This property is crucial for several applications, including drug delivery systems, hydrogels, environmental remediation, and other advanced materials.

One gram of the encapsules was immersed in about one liter of deionized water. After an adequate period, the solution was filtered to separate microcapsules from the solution. The microcapsules were gently wiped using tissue to remove any remaining liquid in encapsule surface and were then weighed to determine their mass in swollen state [52]. Their WSC was calculated using the next formula:

WSC, % = [(Ws – W_o_)/W_o_] × 100

Ws: The weight of microcapsule in swollen state. W_o_: The initial weight of microcapsules.

Volumetric shrinkage (VS) refers to the reduction in encapsules’ volume as they undergo changes in phase, temperature, moisture content, or curing conditions. It was determined based on saturated volume of the encapsules as follows:

VSE, % = [(V_ad_ – V_od_)/V_od_] × 100

V_ad_: Air-dried certain volume of encapsules. V_od_: Oven-dried volume of the encapsules.

#### 2.5.2. Bioassay Screening of the Encapsules Against Termite Control

**Figure 11:**
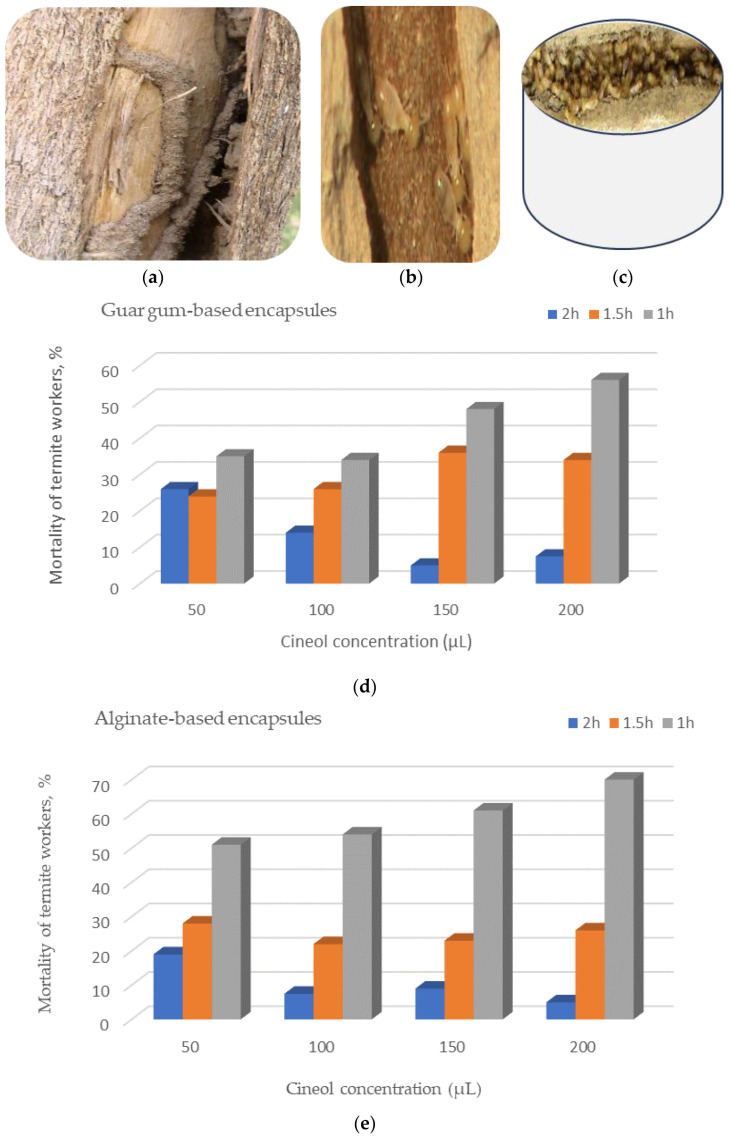
Bioassay screening of the polymeric encapsules uploaded with 1,8-cineol against termite infection: (**a**) infected tunnels fabricated within a *Ziziphus spina-christi*’s trunk, (**b**) a close-up image of alive termite workers, (**c**) a bulk of dead insects after being espoused to cineol within a well-ventilated discant, and (**d**,**e**) the mortality of termite workers after the cineol treatments with guar gum- and alginate-based polymeric encapsules, respectively.

Termite workers were collected among the most common wood in the Hada Al-Sham region, namely, *Ziziphus spina* var. *Christi* (ceder trees). First, ceder woody blocks of about 20 × 6 × 2 cm^3^ were prepared according to Alavijeh et al. [53]; then, they were put in an infested soil. The termites were then separated using a brush and placed in a well-ventilated container (Figure 11a–c) with filter paper that was saturated with distilled water to provide hydration and nourishment. Prior to biometric experiments, the plates were in the dark for 24 h at 28 ± 2 °C and 85 ± 5% relative humidity to reduce termite stress. In order to study the difference between the two polymeric encapsules, namely, guar gum-based encapsules (GGBEs) and alginate-based encapsules (ABEs) uploaded with a natural pesticide, 1,8-cineol was chosen in four different concentrations (50, 100, 150, and 200 μL) for a duration of 1, 1.5, and 2 h. Concentrations of the 1,8-cineol applied for the trials (50, 100, 150, and 200 μL) were prepared using methanol [52,53,54].

Mortality was recorded after each duration, and the traits were repeated three times to represent different replicates.

##### Statistical Work

Two statistical designs were conducted in the present study. The first experiment was conducted as a split-plot design in three replicates to explore the extraction methods’ efficiency of the essential oils obtained from Eucalyptus hybrids grown at three locations (Table S2). Moreover, the second experiment was designed to be split-split plot, one in three replicates for microencapsulation target (Table S3). The purpose of this study was to identify and analyze the variations among the essential oils derived from Eucalyptus leaves using the MASD and ESD technique. Furthermore, the statistical analysis used the least significant difference at a 95% level of confidence approach to assess and compare the variations among the means of different species for all the attributes under investigation [80].

## 3. Results and Discussion

**Figure 4:**
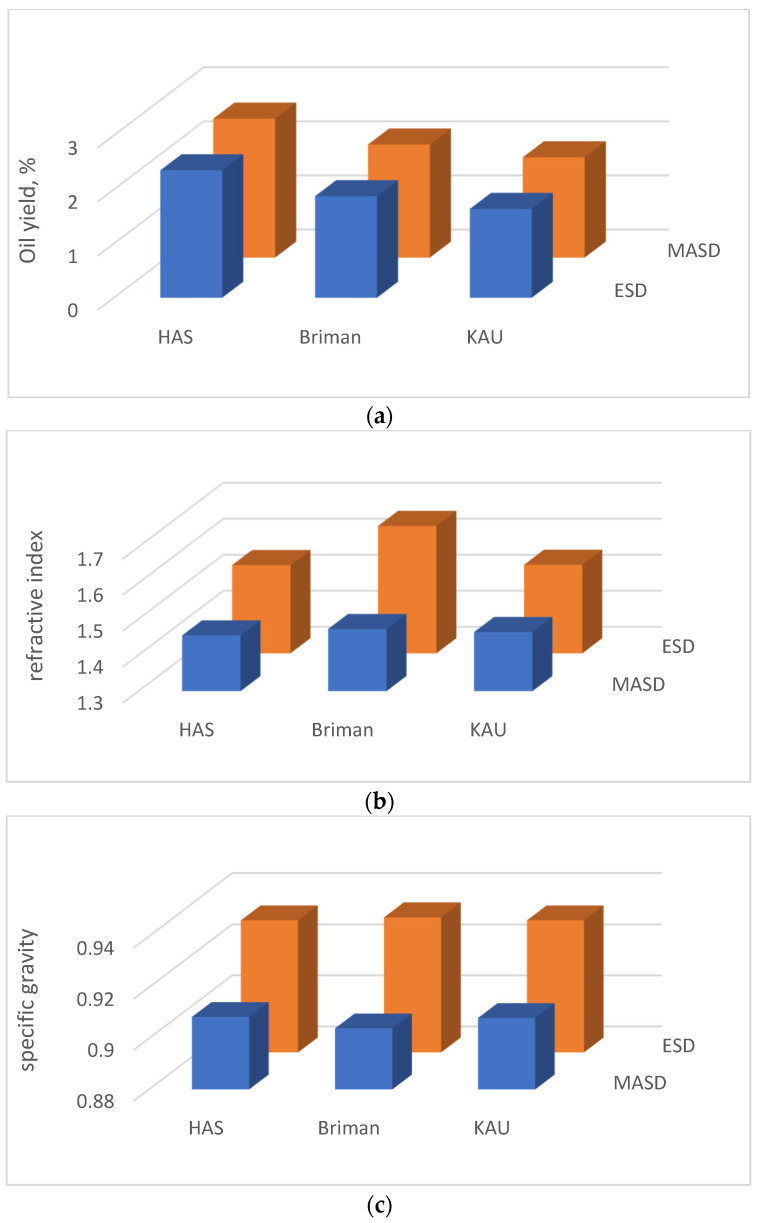
Physical properties of the Eucalyptus’ essential oil extracted using microwave-assisted steam distillation (MASD, the blue histograms) and electric steam distillation (ESD, the orange histograms) from the three *Eucalyptus* ecotypes’ sites: (**a**) oil yield, (**b**) refractive index, and (**c**) specific gravity.

**Figure 5:**
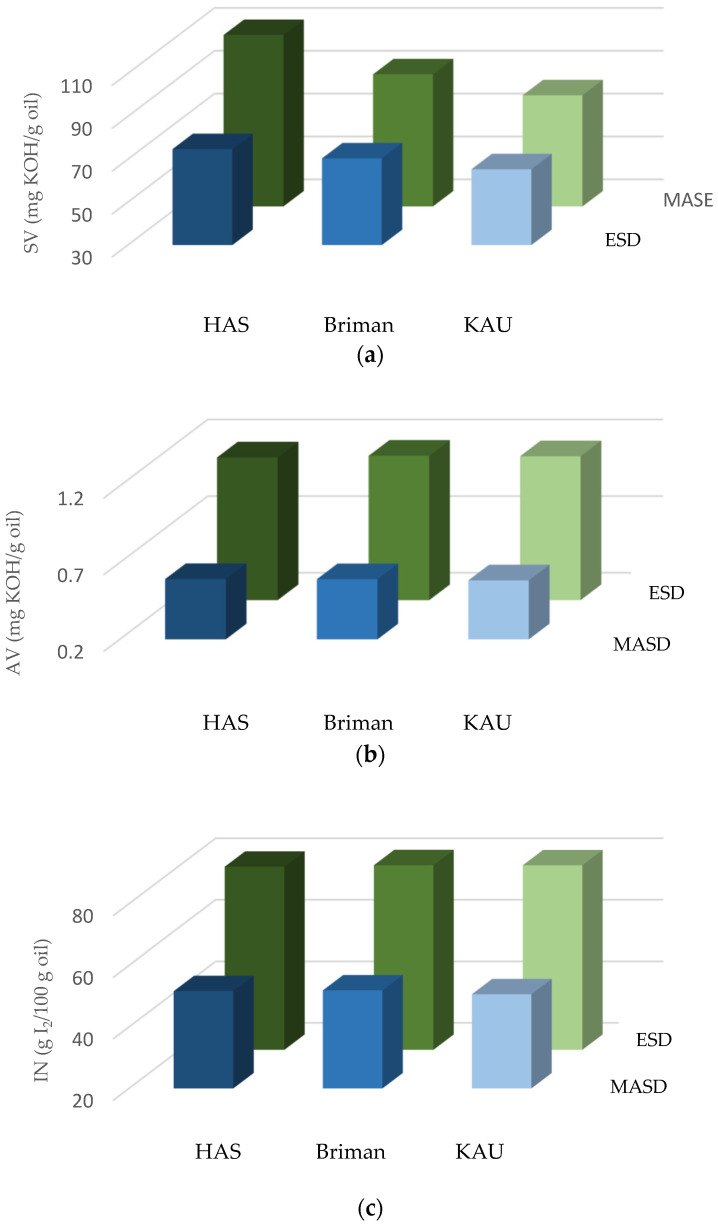
Chemical properties of the Eucalyptus essential oils: (**a**) saponification value (SV, mg KOH/g oil), (**b**) acid value (AV, mg KOH/g oil), and (**c**) iodine number (IN, g I_2_/100 g oil) extracted using each of the microwave-assisted steam distillation (MASD, the blue histograms) and electric steam distillation (ESD, the green histograms) techniques from the three *Eucalyptus* ecotypes’ sites, namely, Hada Al-Sham (HAS), Briman, and King Abdulaziz University (KAU) campus, where the interaction between extraction methods and locations is significant.

**Figure 6:**
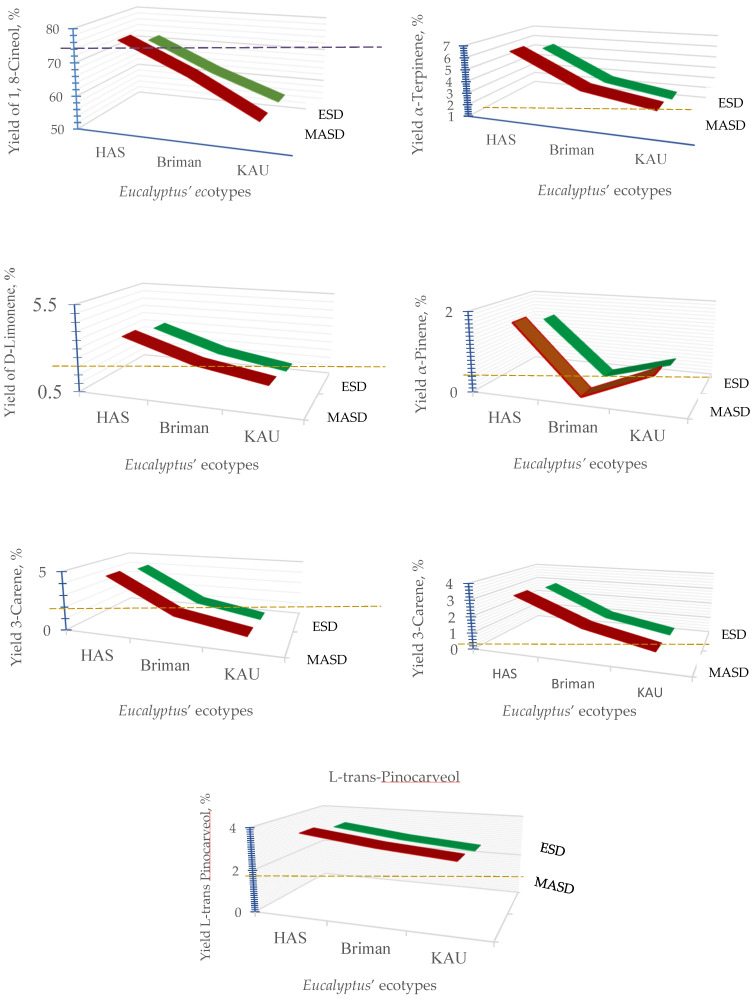
Yields of chemical constituents of the essential oil extracted from different *Eucalyptus* ecotypes using microwave irradiation technique (MASD, the red curve) and electric heating tool (ESD, the green curve) compared with the reference species “*Eucalyptus globulus* Labill” detected at Briman region that are leveled by the red dotted line.

**Figure 7:**
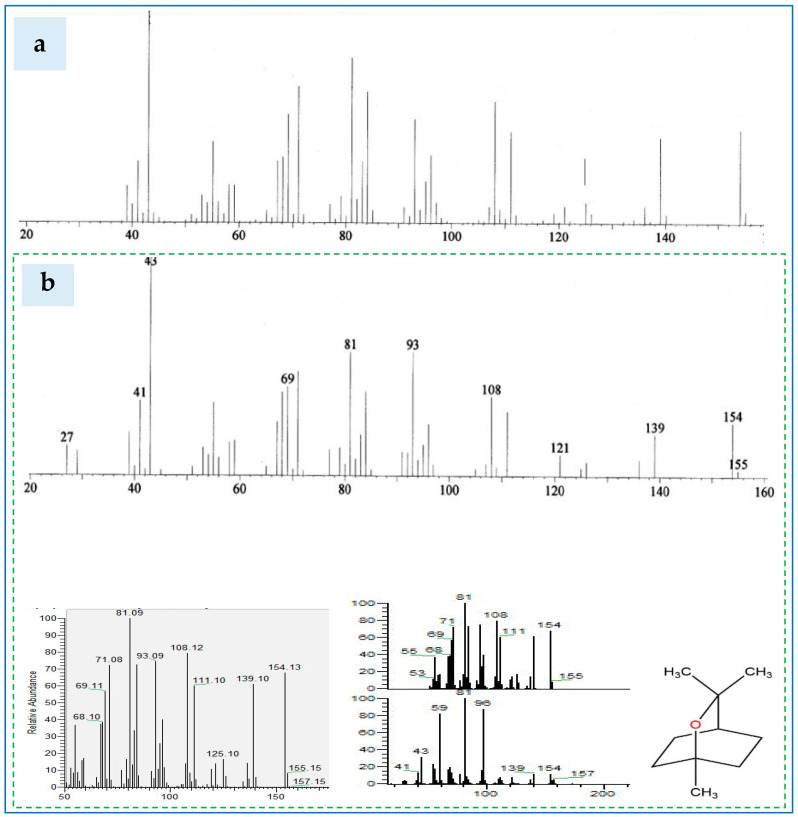
The mass spectra (MS) of 1–8, cineol fractionated from the EEOs extracted from: (**a**) *Eucalyptus globulus* Labill leaves from NIST-02-library’ s GC-MS system, and (**b**) Eucalyptus ecotypes in the current investigation.

The eucalypt essential oils (EEOs) were extracted from the ecotypes grown at each location of Hada A-Sham (HAS), Briman, and King Abdulaziz University (KAU) campus by using each MASD and ESD apparatus. Their physical qualities (oil yield, refractive index, and specific gravity) were investigated and are presented in Figure 4. In addition, their chemical properties are shown in Figure 5, Figure 6 and Figure 7.

### 3.1. Physical Properties of the Essential Oils

#### 3.1.1. Essential Oil Yield (EOY)

Based on the data presented in Figure 4a, it is evident that the EOY varies among the three different locations studied (HAS, Briman, KAU) when employing each of the heating techniques (MASD or ESD). Specifically, the analysis reveals that HAS leaves exhibit the highest yield, with values of 2.57% and 2.36% for MASD and ESD, respectively. The yield obtained from Briman leaves was lower, measuring 2.09% and 1.88% for MASD and ESD, respectively. Similarly, KAU leaves yield even lower amounts of essential oil, with values of 1.85% and 1.64% for MASD and ESD, respectively. Based on the analysis of the Eucalyptus species investigated in this work, it is evident that the MASD process demonstrates high efficacy in extracting essential oil with superior quality while maintaining the integrity of bioactive ingredients due to obtaining homogeneous heating atmosphere as well as precise heating rate. compared to that obtained by ESD. 

The extraction process used in producing essential oils directly impacts the oil’s biological content and functionalities. Factors such as plant diversity, physical conditions, and harvest timing also affect the oil’s quality and quantity [81,82,83,84,85,86,87].

#### 3.1.2. Refractive Index (RI)

Statistical analysis revealed significant variations in the essential oil’s RI values across the different eucalypt ecotypes introduced into the three locations examined using each of the two distillation procedures as shown in Figure 4b. The RI values within the location factor indicates that Briman leaves exhibited the highest index values at the MASD and ESD methods (1.47 and 1.65, respectively) compared to those obtained from Hada Al-Sham (1.45 and 1.55, respectively) and KAU (1.46 and 1.55, respectively (Figure 4)). In general, the ESD yields higher results than the MASD for species with similar levels of oiliness (Figure 4). The RI of Briman leaf oil (1.47) falls within the range of values reported by the study of [88]. However, it was observed to be greater than the range when the oil was extracted using the ESD technique. This finding suggests that using MASD did not result in any significant variation in the refractive index (RI) of the essential oils obtained. This outcome validates the appropriateness of the current research for its intended industrial implementation.

The results of the RI were found within the normal scale found by several studies [66,81,89]. The ESD measurements show elevated values (ranging from 1.55 to 1.65) in relation to the various species obtained from the Hada Al-Sham, Briman, and KAU locations. The greater RI value may be attributed to the elevated temperatures created by the ESD approach. The biological content of essential oils may be influenced by the extraction process used during their manufacturing [88]. The variability in high temperatures throughout the extraction process significantly impacts the quality of the fundamental component. The use of the water distillation process at temperatures over 100 °C for extracting oil from *Eucalyptus camendulis* leaves has been seen to decrease the quantity of oil obtained and may impact the RI as referred by Javid et al. [90]. When Briman leaf oil was tested, it was found that the most significant RI value observed was 1.47, which is within the permissible range of values. It can be inferred that the MASD approach yielded comparable results to those obtained using the same amount of oily species. Consequently, it can be concluded that the MASD method did not introduce any discernible variations in the refractive index of the resulting essential oils [62].

#### 3.1.3. Specific Gravity (SG)

Statistically significant variations in SG values were observed across the Eucalyptus species, which were impacted by the extraction techniques used and their combinations. However, no significant variations were found between and within the species (Figure 4c). Using the MASD technique to compare the SG values among the three locations considered reveals that Eucalyptus leaves provide the lowest value (0.90 in Briman and 0.91 in another two species), whereas ESD gives the most excellent value (0.93 in all three species). The average specific gravity value obtained in the current experiment using both of the approaches falls within the range of 0.957–0.968 specified by the ASTM [71] as reported by the study of [62]. The MASD and ESD procedures provide similar results for oily species at the same level (Figure 4c). It is suggested that both approaches do not alter the resulting essential oils’ specific gravity, highlighting this innovative study’s economic significance.

Various extraction processes and physical circumstances were reported to influence the quality and amount of essential oil production [84]. SG determination in essential oils has significant importance as it serves as a valuable indication of their purity and enables the differentiation of various oily solutions, as noted by the study of [66]. They also noticed that, when an oil spill happens, the chemicals released into the water have a higher specific gravity than the oil itself. The use of the microwave-extraction method in this investigation resulted in the presence of contaminants within the Eucalyptus leaf oil, which might perhaps account for the slight variations seen in the specified results [62,66].

### 3.2. Chemical Properties of the Essential Oils

Regarding the saponification value (SV) of the EO, statistical analysis revealed that the factors studied (extraction methods, locations) as well as their interaction significantly influenced the SV, as shown in Figure 5a. The results revealed that leaves collected from Hada Al-Sham had the highest SV for MASD and ESD (112.64 and 74.80, respectively) compared to the other locations, namely, Briman (91.65 and 70.46, respectively) and KAU (81.86 and 65.41). Among all the sources, leaves collected from Hada Al-Sham contain a higher SV, followed by Briman and KAU under the extraction methods mentioned. In addition, MASD produced approximately 50.59% more SV in the essential oil collected from Hada Al-Sham compared to the action of the ESD.

The EO results showed diverse SV results, with the extraction process, plant components, species variety, and harvest timing all contributing to the yield and quality of the EOs, potentially influencing its biochemical composition and functionality [80,81,82,83]. It was reported that the SV correlates the molecular weight (MW) of triglycerides, with higher SVs corresponding to lower MW values [62,66]. Consequently, it was anticipated that EO would possess a reduced molecular weight of triglycerides compared to other EOs exhibiting an SV. The results shown in Figure 5 and Figure 6 indicate that the SV of EO fell within the ASTM-mean value [72] range of 175–187 SV units, demonstrating its high SV. This finding is consistent with other studies published [66,89,91,92].

The analysis of the acid value (AV) indicated that the extraction methods (MASD and ESD) had a notable impact on the EO. However, the influence of within eucalypt’s ecotype variations and their interaction was statistically insignificant (Figure 5b). Using the ESD technique, the Eucalyptus leaves obtained from the Briman and KAU regions had the greatest acid value (AV) of about 1.14 mg KOH/g oil. This AV was found to be 93.22% higher compared to the AV of the Eucalyptus leaves collected from the Hada Al-Sham and Briman regions, which had an AV of around 0.59 mg KOH/g oil (Figure 5b). Both approaches yielded comparable findings for the Eucalyptus’ ecotypes, with values ranging from 0.58 to 0.59 mg KOH/g oil in the MASD method and 1.13 to 1.14 mg KOH/g oil in the ESD method.

In the current investigation, the highest acid value (AV) of the essential oil (1.14 mg KOH/g oil) falls within the specified range of [72] (0.4–4.0 mg KOH/g oil), as shown in previous studies [66,93]. The Eucalyptus oil had a low concentration of free fatty acids, as shown by the acid value (AV), which indicates the carboxylic acid groups present in the fatty acids comprising the oil.

Additionally, the AV quantifies the quantity of free fatty acids (data not provided). The present investigation yielded a lower average value (AV) for EOs compared to the AV for castor oil seed determined previously by Hindi et al. [62]. They state that the seeds were gathered from the ground and underwent a curing process for a specific duration. Subsequently, the seeds were subjected to an adequate quantity of lipase enzyme, facilitating the hydrolysis of their triglycerides into free fatty acids. This enzymatic process increased the seeds’ acid content.

In relation to the iodine number (IN), a notable observation was noticed regarding the MASD and ESD methods applied to Eucalyptus essential oil. Specifically, the Briman species exhibited a higher IV of 80.04 g I_2_/100 g of oil, while the Hada Al-Sham species had an IV of 79.50 g I_2_/100 g of oil, and the KAU species had an IN of 79.99 g I_2_/100 g oil (Figure 5c). Similarly, in the ESD method, the Briman species had a higher IN of 51.91 g I_2_/100 g oil, compared to the Hada Al-Sham species with an IN of 51.65 g I_2_/100 g oil and the KAU species with an IV of 50.63 g I_2_/100 g oil. Moreover, the IN values acquired with the MASD approach yielded greater INs compared to the species recovered using the ESD technique. Furthermore, when considering both intra-species and combinations, no statistically significant variations were seen among the various investigated species (Figure 5c). The IN values for all species (ranges 50.63 to 51.91 g I_2_/100 g of oil) were lower than the 100 IN unit for ESD, as Figure 5c illustrates, but they are still within the ASTM [70]-specification limit (82–88 g I_2_/100 g of oil). Conversely, MASD’s highest IN readings (79.50 to 80.04 g I_2_/100 g of oil) were all lower than the standard range for all species.

The lower iodine number (IN) readings may be ascribed to the higher concentration of saturated fatty acids that did not undergo a chemical reaction with the Hanus iodine solution [66]. The Eucalyptus species exhibited potential for producing high-quality essential oil in this study. It is attributed to the higher IN values observed, which fall within the specified ranges outlined by ASTM [72]. The elevated levels of unsaturation indicated by the values suggest a greater capacity for unsaturated acids to absorb iodine. Consequently, EOs derived from ESD possesses characteristics that make them well suited for use as non-drying oils in the cosmetic industry. However, their compatibility with the paint industry may be limited, while their suitability for the soap industry remains favorable [94]. Since unsaturation is directly related to the IV and the RI, the oil’s moderate RI value aligns with its reasonable iodine number [62].

### 3.3. Chemical Constituents of the Essential Oil

The volatile chemical composition of essential oil was investigated using GC/MS. Seven significant chemicals were identified, which mainly consisted of 1,8-Cineol, α-terpinene, D-limonene, α-pinene, 3-carene, α-myrcene, and *L-trans*-pinocarveol. The compounds constitute the majority of the total essential oil (Figure 6 and Figure 7).

The EO extracted from the leaves of Eucalyptus hybrids grown at the three different sites differed at their chemical composition. Their differences may be attributed to the fact that the trees grown in different regimes may exhibit differences in their chemical constituents [68] as well as their botanical difference.

The MASD and ESD procedures were shown to be appropriate for extracting some of the key compounds present in Eucalyptus leaves. It is evident that one of the main constituents was 1,8-cineole, which is often referred to as eucalyptol. Previous studies showed comparable compositions of the EOs derived from the Eucalyptus variety [95]. The studies identified α-pinene (17.45%), β-pinene (0.28%), 4-terpineol (0.33%), and spathulenol (1.87%) as the predominant constituents of the EOs. The compound 1,8-cineole plays a crucial role in determining the economic worth of the oil and its importance as a primary resource for many businesses. Various studies have shown varying amounts of 1,8-cineole in the leaf oil of Eucalyptus globulus, ranging from 47% to 87%, across various nations [96,97].

The extraction techniques, as well as the specific *Eucalyptus* ecotypes and their combined effect, substantially impacted the chemical makeup of the extracted essential oils (Figure 6). The findings of this study indicate that the volatile oil derived from each species of Eucalyptus has a distinct chemical makeup in terms of quantity and quality. As per the findings, the most prevalent chemical was eucalyptol (1,8-cineole), constituting the highest proportion. Nevertheless, many additional compounds were relatively abundant, including α-terpinene, D-limonene, 3-carene, and *L-trans*-pinocarveol. On the other hand, α-Pinene and α-Myrcene were identified as less abundant compounds (Figure 6 and Figure 7).

The volatile oil derived from Eucalyptus leaves is mostly composed of a higher ingredient commonly found in most Hada Al-Sham ecotypes. However, there are variations seen in the quantities of some essential oil molecules among the species. The observed variances in the mentioned phenomenon may be attributed to genetic factors [96]. Additionally, geographical and meteorological circumstances have been identified as potential contributing factors, along with other variables like the time of harvest, age of the plant, and the technique of distillation [98,99].

The current results align with previous studies conducted by Tsiri et al. [100] and Cimanga et al. [101], which demonstrated that the primary constituents of Eucalyptus oils were mostly composed of 1,8-cineole. The chemical compositions of many different species of Eucalyptus were documented in a publication by Batista-Pereira et al. [102], and the findings are consistent with the results obtained in this study. The observed variation might perhaps be attributed to disparities in the chemical composition of the plants. Previous research has corroborated the current results of this study [103].

The majority of the chemicals classified as monoterpene alcohols comprised the predominant class within the overall oil composition. In a previous study, the authors of [104] identified oxygen-containing monoterpenoids as the predominant constituents in 20 distinct kinds of Eucalyptus oil. The chemical ingredients of the EEO isolated in this research have potential use in medical therapy. Eucalyptol, also known as 1,8-cineol, was identified as a predominant component (44–84%) in the essential oil derived from several species of Eucalyptus. Significant research conducted by Sebei et al. [97] and Sahi [105] has shown the noteworthy antioxidant and antibacterial properties associated with this chemical. Furthermore, it is worth noting that the Eucalyptus essential oil has a significant proportion of eucalyptol, around 70%, as indicated by the European and British Pharmacopoeia [106]. This particular composition makes it highly suitable for employment in therapeutic applications.

One significant monoterpene ester identified in the EO is α-terpinyl acetate. The substance has antibacterial properties and possesses a pleasant scent characterized by a sweet, flowery floral and lavender aroma [88]. Therefore, α-terpinyl acetate is extensively used as a significant component in air fresheners, laundry detergents, dishwashing solutions, deodorizers, soaps, shampoos, and lotions. In addition, α-terpinyl acetate serves as a food flavoring ingredient in various baked goods, beverages, fruit-based ice creams, sugar confections, chewing gum, gelatin-based products, and puddings [107].

It is clear that 1,8-cineole (eucalyptol) was the primary constituent in all the species obtained from Hada Al-Sham, Briman, and KAU using both extraction procedures. The leaves obtained from Hada Al-Sham have a high concentration of 1,8-cineole, with a content of 76.15% as determined by MASD and 72.15% as determined by ESD. In contrast, MASD and ESD methods yielded extraction efficiencies of 67.12% and 63.12% (Briman) and 56.19% and 55.85% (KAU) for the compound 1,8-cineole, as shown in Figure 6.

The extraction procedures had a substantial impact on the components α-terpinene and 3-carene across all Eucalyptus species; however, their combined effect was found to be non-significant (Figure 6). The leaves obtained from Hada Al-Sham exhibited the highest levels of α-terpinene (6.42% and 5.59%) and 3-carene (4.51% and 4.14%) when analyzed using the MASD and ESD techniques, respectively. D-limonene (3.60%) and *L-trans*-pinocarveol (3.66%) were found to be present in significant quantities as major constituents in the species obtained from Hada Al-Sham (Figure 6).

Additionally, α-pinene and α-myrcene comprise a small but significant portion of the oil’s overall composition. Briman and KAU species have very low concentrations of them. The species taken from Hada Al-Sham exhibited the highest concentrations of α-pinene (1.69%) and α-myrcene (3.18%) among all samples, regardless of the extraction technique used (Figure 6). The MASD approach generally has more promise for quantifying the volatile component of the essential oil derived from Eucalyptus leaves.

The total-ion chromatogram of 1, 8-cineole in the essential oil of *Eucalyptus globulus* Labill leaves as compared to that from NIST-02-library data of the GC-MS system is shown in Figure 7. It is worth mentioning that the ratio of an ion’s mass (m) in atomic mass units (amu) to its formal charge (z) was estimated and was presented as the X-coordinate. Examining Figure 7a,b revealed that the cineole detected in *Eucalylus globulus* was featured by MS’ curves ranged from 10 to 157 *m*/*z*. On the other hand, the EEOs resulted from the hybrids’ ecotype were found to contain *m*/*z* chromatograms approaching to those detected in *Eucalyptus globulus* (ranged from 10 to 157 *m*/*z*). This finding indicates the suitability of examined Eucalyptus’ ecotypes as a source of 1,8-cineole that is useful for various midicinal and food industries.

### 3.4. Microwave Theory Operation

**Figure 8:**
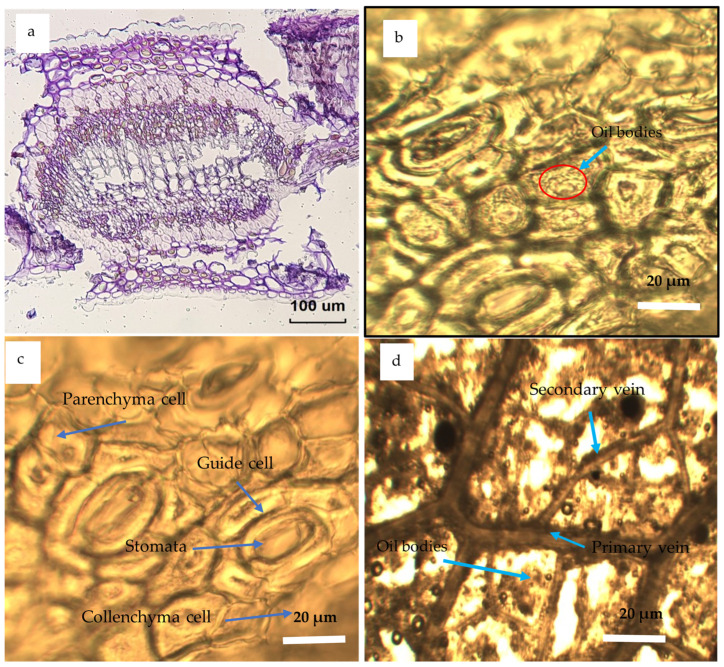
Optical micrographs of anatomical structure of the Eucalyptus leaves collected from the three ecotypes showing (**a**) overall transverse section, (**b**) oil bodies, (**c**) parenchyma cells, guide cells, stomata and collenchyma cells, and (**d**) secondary veins, oil bodies, and the primary vein.

**Figure 9:**
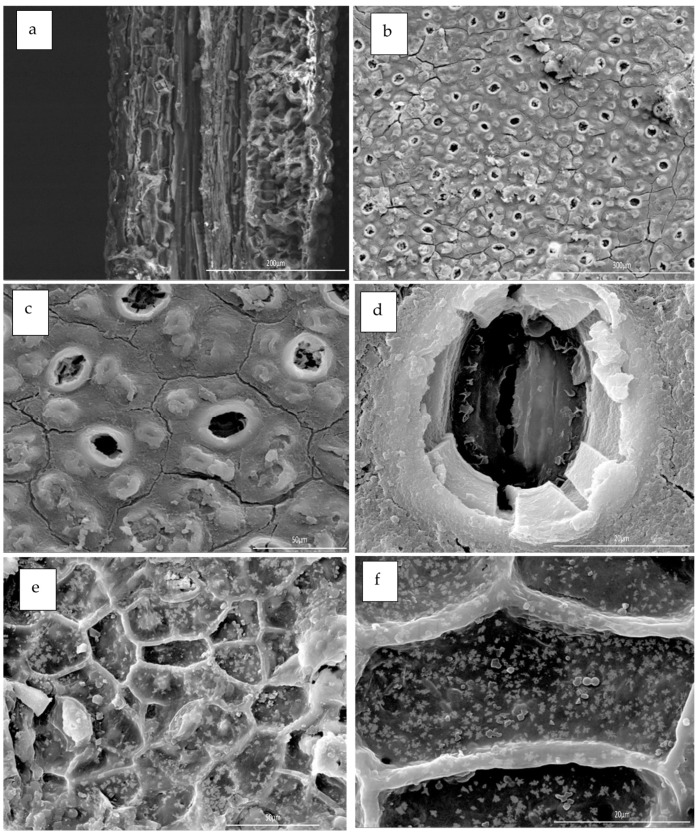
Scanning electron microscopy (SEM) micrographs of anatomical structure of the Eucalyptus leaves collected from the three ecotypes shown in circles: (**a**) guide cells, (**b**) oil bodies, (**c**) stomata, (**d**) parenchyma cells, (**e**) collenchyma cells, (**f**) secondary veins, and oil bodies.

In order to investigate and illustrate the impact of microwave irradiation on biopolymeric structured tissues, an anatomical examination of the leaf precursors was conducted using scanning electron microscopy (SEM). The results of this examination are shown in Figure 8 and Figure 9 for optical and SEM micrographs, respectively [108,109,110,111,112,113].

It is worth mentioning that Figure 2 shows the electric- and microwave-assisted steam distillation apparatus used for extracting the essential oil from leaves of eucalypt ecotypes. Moreover, Figure S1 provides technical details pertaining to the microwave irradiation equipment that has replaced conventional electric heating coils in a traditional autoclave apparatus used for extracting the eucalypt EO. The provided illustration depicts the components of the microwave generating unit (MGU) as shown in Figure S1a that is constituted to a high-voltage transformer (Figure S1b) together with the high-voltage magnetron (Figure S1c) and a high-voltage capacitor (Figure S1d). The method was used to direct the microwave beam to the distillation vessel through a dedicated waveguide.

Microwave wavelengths cover a range of about one meter to one millimeter, exhibiting frequencies that vary from 300 MHz (1 m) to 300 GHz (1 mm). Electromagnetic waves have distinct wave properties, although they also manifest particle properties when seen at high frequencies. [62]. Academics are exploring microwave irradiation for oil extraction from plants using a high-voltage transformer, which operates at a voltage of 220 V and a frequency of 60 Hz [62,108,109,110,111]. To achieve the desired frequency of 2.45 GHz, the magnetron undergoes a conversion process whereby it transforms high-voltage alternating current (AC). According to reports, magnetrons operating at a frequency of 915 megahertz are used in industrial and commercial ovens to stimulate the bigger cavities present inside the ovens [61,62,75].

It is worth mentioning that heat can be transferred by one or more of a triple motion phenomenon (conduction, convection, and radiation) that facilitates heat transfer from the outer regions of leaf tissues to their inner cores. Heat transfer by conduction is controlled by Fourier’s law, and heat transfer by convection is governed by Newton’s law, and/or by radiation, which is regulated by Kirchhoff’s law.

Microwave beams, transmitted through leaf tissues, absorb microwave radiation, generating an oscillating electric field. This results in molecular rotation and alignment, generating heat. This absorption leads to intense vibrations, causing friction, which increases temperature, facilitating efficient processes like water evaporation, fat melting, and EO evaporation.

Therefore, there is a significant augmentation in the vapor pressure gradient between the innermost region and the outer surface of the biopolymeric tissue, hence facilitating the rapid diffusion of moisture and/or essential oil from the tissue. Therefore, it can be inferred that microwave drying and microwave hot-pressing techniques exhibit superior characteristics in terms of speed, uniformity, and energy efficiency when compared to conventional methods [62,112].

Electromagnetic waves, characterized by their electric and magnetic fields (Figure A3a,b), have the capability to introduce energy into a given system [60,61,74]. The generation of thermal energy occurs due to molecular rotation, when molecules collide with one another and transfer kinetic energy, initiating their motion. Liquid water is the most effective medium for microwave heating due to its high efficiency. In contrast, substances such as fats and sugars, which possess less molecular dipole moments, as well as frozen water, where molecular rotation is restricted, exhibit lower efficiency in this regard.

Fields exerted force and move charges in systems, enhancing energy transmission when electromagnetic waves align with the system’s frequency. Energy is proportional to amplitude, and greater electric and magnetic fields impose pressures. However, microwave beams have temperature variations (hot and cold spots), making them inappropriate for heating the extraction vessel. It is worth mentioning that microwave beams can create hot spots alternated to cold (damping) spots in eucalypt’ s leaves tissues present at the extraction vessel reducing heating efficiency. This defect is apparent from examining the propagation line (the baseline) of the microwave sinusoidal curve shown in Figure A3a–c. In order to solve this issue, the botanic tissue was allowed to be rotated by a novel manner by using an innovative gaseous agitation device used to rotate the evaporated water steam-bearing oils. This innovative device improved heating efficiency by exposing tissues to the microwave beams from different angles, which helps to ensure that all parts of the leaves receive sufficient energy. This is particularly important for larger or irregularly shaped items that may not heat evenly otherwise. Furthermore, for illustrating the dangers of hot spot stability in the extraction process, it can be indicated that, if the tissues remained stationary, certain areas may absorb more microwave energy, leading to hot spots, where some parts are overly heated or even burnt, while others remain cold. The rotation helps mitigate this effect. Accordingly, the undesired hot spots’ effect was illuminated by applying this invention [113].

Regarding the novel gaseous agitation invention, its ability to rotate the water steam within the extraction vessel, allowing all leaves’ charge to excrete their content of essential oil more efficiently, is presented in Figure A3d. Four pipes’ inlets were centrally inserted within the extraction vessel passing through the cover plate. Each pipe was connected to an electric solenoid that controls its opening. The four solenoids are connected in parallel with programmable solenoid valves. Specifically, when water evaporated due to the microwave effect, the emitted steam was entering one pipe that was opened by a working solenoid through a programmer controller and driver. The programmer permits one solenoid only to be opened, while the other three solenoids are closed. This process was repeated subsequently and consequently. This can allow the released water steam to be rotated and the plant tissues to be heated homogeneously without causing permanent alternated hot and/or cold spots [113].

The MASD machine is an optimal instrument for facilitating heat transmission. Consequently, the use of this machine to heat the leaf tissues was shown to enhance the EO production, quantitively and qualitatively as supported by previous studies [114] as well as the current one.

### 3.5. Effect of Microwave Irradiation on the Leaves’ Tissues Bearing the Essential Oil

**Figure 10:**
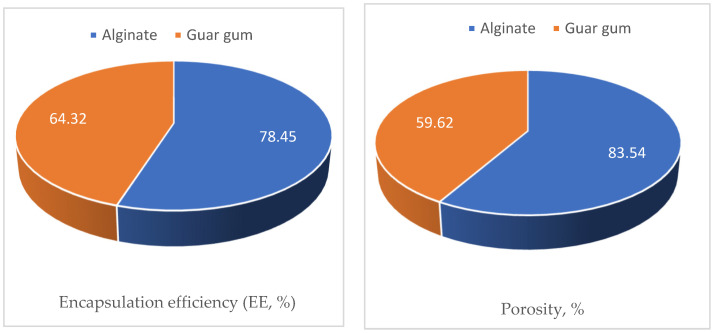

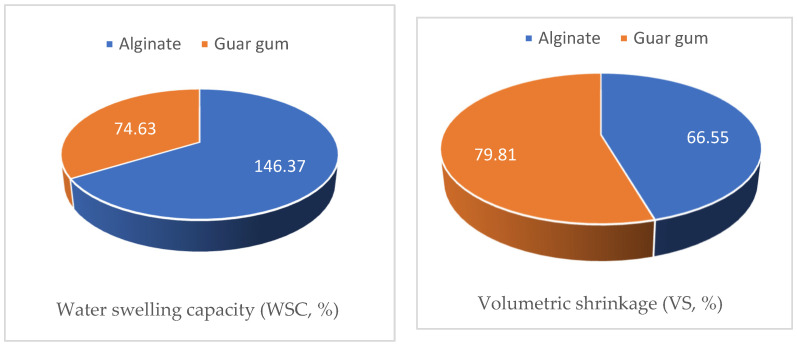
Mean values of the physical properties of the polymeric encapsules fabricated from each of the guar gum and alginate and uploaded with 1,8-cineol.

Studying and understanding microstructure of various organelles found in leaf tissue is beneficial in enhancing the efficacy of essential oils (EOs), characterization processes, as well as in industrial processing, particularly for assessing the feasibility of substituting microwave irradiation with conventional heating methods in oil extraction process [115]. In contrast to traditional techniques, the use of microwave treatment for oil extraction has several benefits. The advantages include enhanced yield and quality of the extracted oil, the capacity to directly extract oil, decreased energy consumption, accelerated processing time, and reduced solvent contents [62,75,109]. The observed outcomes may be ascribed to the use of microwave irradiation, which presents a promising option for inducing stress responses in the highly organized tissues seen in oil seeds. The use of microwave radiation on oil extractions has been shown to generate greater extraction rates and enhanced mass transfer coefficients due to the more pronounced rupture of the cell membranes. In addition to this, permanent pores were formed in a manner consistent with the movement of the EOs through permeable cell walls [93,116]. Optical and SEM’s histological features of the *Eucalyptus globulus*’ leaves are shown in Figure 9 and Figure 10, respectively. These microscopic units are several anatomical features of the Eucalyptus leaves collected from the three ecotypes showing the following: (a) overall transverse section, (b) oil bodies, parenchyma cells, guide cells, stomata, collenchyma cells, primary and secondary veins, and oil bodies.

### 3.6. Characterization of the Microcapsules

Encapsulation efficiency (EE), porosity, water swelling capacity (WSC), and volumetric shrinkage (VS) of the guar gum and alginate skeletons were investigated as presented in Figure 10.

The obtained porosity data presented in Figure 10 indicated that alginate encapsules had higher porosity values compared to the guar gum ones (83.54% and 59.62%, respectively). This finding illustrates the superior behavior of the alginate encapsule in controlling termites can be attributed to the higher uploading of the cineol as a result of its higher porosity compared to the guar gum encapsules.

### 3.7. Bioassay Screening of the Encapsules Against Termite Control

Infected tunnels fabricated by alive termite workers within a *Ziziphus spina-christi*’s trunk is presented in Figure 11a–c. Moreover, the encapsulation process’ results was performed to study the bioassay screening of the polymeric encapsules (guar gum- and alginate-based encapsules (GGBEs and ABEs, respectively), which were uploaded with different concentrations of 1,8-cineol in a concentration of 50, 100, 150, and 200 μL for a duration of 1, 1.5, and 2 h (Figure 11d,e).

Through the bioassay screening task, termite workers were collected among the most common wood in the Hada Al-Sham region, namely, *Ziziphus spina* var. *Christi* (ceder trees). First, the ceder woody blocks of about 20 × 6 × 2 (cm^3^) were prepared according to Alavijeh et al. [53]; then, they were put in an infested soil. The termites were then separated using a brush and placed in a well-ventilated container (Figure 11a–c) with filter paper that was saturated with distilled water to provide hydration and nourishment. Prior to the biometric experiments, the plates were in the dark for 24 h at 28 ± 2 °C and 85 ± 5% relative humidity to reduce termite stress.

In order to study the difference between the two polymeric encapsules, they were prepared using methanol. The termite mortality was recorded after each duration, and the traits were repeated three times to represent different replicates.

Concerning the efficiency of polymeric hydrogels studied as biopesticides for termite control, it is clear from Figure 11d,e that the ABE was more efficient than the GGBE regardless of cineol concentration and the duration exposure. This evaluation was based on the higher mortality of termite workers exposed to the ABE than the GGBE.

Furthermore, regardless of the encapsule type and the exposure duration, the mortality percent of the insects were exceeded significantly for the high cineol concentrations compared to the lower concentrations for both the ABE and GGBE. The higher the cineol concentrations, the higher the mortality percentage of the termites (Figure 11d,e). This finding can be attributed to the rapid toxic effect of the cineol compound at higher concentrations.

Extending to present the pest controlling’s bioassay effort, it can be seen from Figure 11d,e that the time of exposure to the encapsule by the termite workers affected their mortality percent, which permits insects to breathe more bio-insecticide than within shorter periods. Since there is a lot of evidence and results of previous studies indicating the toxicity of the 1,8-cineole compound to many insects, it can be said that, during the first hour, a peak level of exposure occurs, which may trigger immediate mortality. After that, there may have been a reduction in cineol concentration and, subsequently, the exposure effects, possibly due to metabolic processes that detoxify the substance or its absorption into tissues. The outcomes of the present study were aligned with those obtained by other investigations [53,54,55].

**Figure A4:**
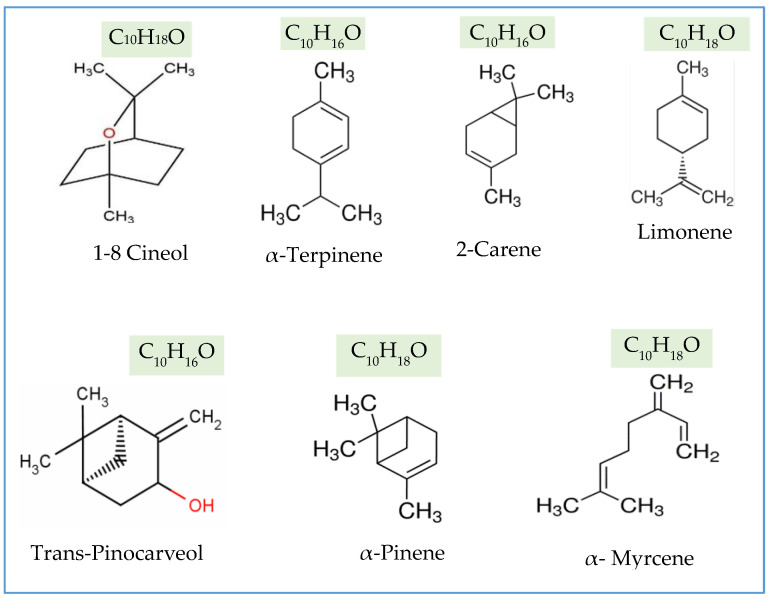
Chemical formulas and chemical structures of the important constituents of the essential oil extracted from leaves of *Eucalyptus* ecotypes.

The oils were fractionated into their noticeable chemical compounds that were compared to those detected in the *E. globulus* Labill and their chemical formula are presented at Figure A4 [117].

## 4. Conclusions

Essential oils (EOs) from *Eucalyptus* ecotypes’ leaves grown in three locations of the Eastern region of KSA were extracted using a novel microwave procedure (MASD) and compared to electric extractors. The extraction methods and the ecotypes influenced the chemical composition of their essential oils. Eucalyptol (1,8-cineole) was the main chemical component in all ecotypes. The extraction methods significantly influenced the concentrations of compounds, with Hada Al-Sham leaves showing the most elevated amounts as well as the overall EO’s yield. It was confirmed that using the MASD, in addition to its beneficial properties of being simple, facile, more ecofriendly, and cost-effective, kept oils true to their original form and allowed the warming of larger machines and spaces. Concerning the biopesticides’ encapsule efficiency for termite control, using alginate was more efficient than that fabricated from guar gum regardless of cineol concentration and duration exposure due to higher porosity of the former. This finding can be attributed to the fact that higher porosity allows higher uploading of the biopesticide cineol into permeable hydrogels. Moreover, the time of termites’ exposure to cineol affected their mortality due to breathing more cineol in long periods than shorter ones. Regardless of the encapsule type and the exposure duration, termites’ mortality exceeded significantly for the high cineol concentrations than the lower ones for both encapsules’ materials. This finding can be attributed to the rapid toxic effect of the higher cineol concentrations. During the first hour, a peak level of exposure occurs, which may trigger immediate mortality. After two hours, there may have been a reduction in exposure effects, possibly due to the metabolic processes that detoxify the substance or its absorption into tissues.

## 5. Future Perspectives

More studies are required for the possible commercial uses of essential oils extracted from various Eucalyptus species concerning their medicinal potential and may include looking into their antibacterial, antioxidant, or other therapeutic characteristics as well as biopesticides. The action mechanism of cineole for termite control should be studied in relation to the bioassay screening of the encapsules, decreasing the need for artificial pesticides in their optimal composition, concentration, and/or its reinforcement with additional natural substances.

## 6. Patents

Fenton reactor with gaseous agitation (US Patent no. 11136715B1); microwave-assisted extraction of fixed oils from seeds (US Patent, application No. 63445512).

## Figures and Tables

**Table 1:** The GC-MS settings utilized to analyze the essential oils.

Parameter			Value
GC	Column dimension	Length	30 m
		Internal diameters	0.25 mm
		Solvent’s thickness film	0.25 μm
	Column temperature	Initial temperature (IT)	40 °C
		Residence time (RT) of the IT	4 min
		Maximum final temperature (MFT)	220 °C
		RT of the MFT	15 min
		Heating rate	4 °C/min
	Injector temperature		250 °C
	Injection volume		1 μL
	Flow rate of the carrier gas (helium)		20 mL/min
	Transfer temperature		280 °C
MS	Electron ionization (EI) mode		Negative chemical ionization
	Ionization voltage		70 eV
	Ion source temperature		180 °C
	Scanning range		50–600 Da

## Data Availability

The original contributions presented in this study are included in this article and Supplementary Materials, and further inquiries can be directed to the corresponding author.

## Supplementary Materials

The following supporting information can be downloaded at https://www.mdpi.com/article/10.3390/polym17091182/s1, Figure S1. The electric components of the microwave generator unit (MGU) used for heating the extraction vessel of the MASD: (a) overall image of the MGU, (b) the high-voltage magnetron, (c) the high-voltage transformer, and (d) the high-voltage capacitor; Figure S2. Preparation of methyl esters of the essential oils for analysis by GC-MS; Table S1. Comparisons between extraction methods of essential oils; Table S2. Statistical design of split plot for studying the essential oil extracted from Eucalyptus hybrids grown at each of the campus of King Abdulaziz University (KAU), Hada Al-Sham, and Briman by using two extraction methods, namely, microwave-assisted steam extraction (MASD) and electric-steam extraction (ESD) as repeated for three blocks; Table S3. Statistical design of split-split plot for the encapsulation investigation to study the difference between two polymeric encapsules, namely, guar gum-based encapsules (GGBEs) and alginate-based encapsules (ABEs), which were uploaded with different concentrations of 1,8-cineol in a concentration of 50, 100, 150, and 200 μL for a duration of 1, 1.5, and 2 h as repeated for three blocks.

## Nomenclature

Symbol	Definition
AC	Alternate current
ACS	The American Chemical Society
ADB	Air-dried membrane
AFM	Atomic force microscopy
ANOVA	The analysis of variance
ASTM	American Society for Testing and Materials
AV	Acid value
BST	Biopolymeric structured tissue
CI	Crystallinity index
CLB	Compound lipid body
CW	Cell wall
CY	Cytoplasm
DC	Direct current
DSC	Differential scanning calorimetry
DTA	Differential thermal analysis
EC	Endosperm cells
EHPO	Electric hot-pressed oil
EHPM	Electric hot-pressing machine
ES	Essential oil
ESD	Electric steam distillation
FEG	Field Emission Gun in the SEM
FEI	Field Electron and Ion US Company
FOY	Fixed Oil Yield
FTIR	Fourier transform infrared spectroscopy
GC-MS	Gas Chromatography–Mass spectrometer
GHz	Frequency
HC	Heat change in µVs/mg
HPM	Hot-pressing machine
HVT	High-voltage transformer
IN	Iodine number
LSD	Least significant difference
MAEO	Microwave-assisted extracted oil
MFT	Maximum final temperature
MGU	Microwave generator unit
MHPM	Microwave hot-pressing machine
NDB	Nanodehydrated-bioplastic membrane
NIST	The National Institute of Standards and Technology
NPS	Nanometric particle size
OY	Oil yield
PD	Pore diameter
pH	The acidity or basicity number
PS	Particle size
PubChem	An open chemistry database managed by the National Institutes of Health (NHI)
PVA	Polyvinyl alcohol
RI	Refractive index
RT	Residence time
SD	Standard deviation
SEM	Scanning electron microscopy
SEP	Self-electrostatic peeling
SG	Specific gravity
SLB	Singular lipid body
SOV	Source of variation
SP	Statistical parameters
SR	Surface roughness
SV	Saponification value
SWC	Sinusoidal wave curve
TGA	Thermogravimetric analysis
TR	Temperature range
VFHF	Vibrated-free horizontal flow
VV	Void volume
XRD	X-Ray diffraction

## Appendix E

**Table A1:** Chemical information of the two polymers and their crosslinkers used for synthesis of the encapsules’ hydrogels.

Property		Chemical Compound			
		Polymer		Crosslinker	
		Guar Gum	Alginate	Borax	Calcium Chloride
Chemical structure					
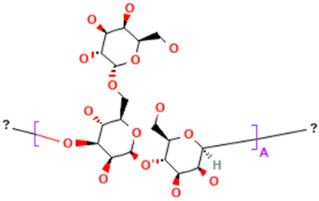
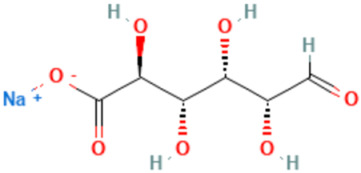
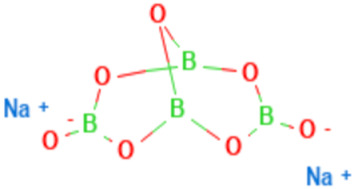
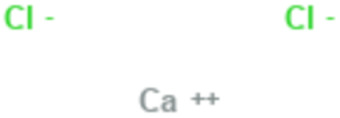
Chemical formula		C_10_H_14_N_5_Na_2_O_12_P_3_	(C_6_ H_7_ O_6_Na)_n_	Na_2_B_4_O_7_·10 H_2_O	CaCl_2_
Molar mass, g/mol		50,000 to 8,000,000	10,000–600,000	381.37	111
Density, g/cm^3^		0.8–1.0	1–1.03	1.73	2.15
Melting point	at 1.013 hPa, °C	>100	99	75	775°
Boiling point		220	495.2 °C	1575	1935
pH (in aqueous solution: 100 g/L, 20 °C)		5.5–6.2	5.5–7.5	9–9.5	8–10
Solubility, g/L		Soluble in hot water (95 °C)	Slowly soluble in water forming a viscous, colloidal solution, practically insoluble in ethanol (96 per cent)	49.74	745
Color		Yellow White	Dark Yellow	White	White
Odor		Odorless	Odorless	Odorless	Odorless
IUPAC name			Sodium 3,4,5,6-tetrahydroxyoxane-2-carboxylate	disodium; 3,7-dioxido-2,4,6,8,9-pentaoxa-1,3,5,7-tetraborabicyclo [3.3.1]nonane	Calcium dichloride
