# Supplementary material for: Fabricating Natural Polymeric Encapsules for Pest Control Uploaded with 1,8-Cineole Extracted from Eucalypt Ecotypes’ Leaves Using Innovative Microwave Tool
Source: Polymers (Basel). 2025 Apr 26;17(9):1182. doi: 10.3390/polym17091182 (PMC12073210; doi:10.3390/polym17091182)
Supplement: Supplementary file 1 [file polymers-17-01182-s001.zip › polymers-3611300-supplementary.pdf]

Supplementary Materials for

Fabricating Natural Polymeric Encapsules for Pest Control Uploaded with 1, 8- Cineole Extracted from Eucalypt Ecotypes' Leaves using Innovative Microwave Tool

Sherif S. Hindi

Department of Agriculture, Faculty of Environmental Sciences, King Abdulaziz University (KAU), P.O. Box 80208, Jeddah 21589, Saudi Arabia; shindi@kau.edu.sa; Tel.: +96-656-676-0086

This PDF file includes the following:

Table S1. Comparisons between extraction methods of essential oils.

Method	Advantages	Disadvantages
Hydro-distillation	Easy to use, selective, and flexible.	It is not feasible to remove everything; the process takes too long and uses too much energy.
Steam distillation	In contrast to hydro-distillation, less time is spent extracting, and fewer polar molecules are lost.	Longer extraction times result in non-appreciable and more expensive chemicals because of the extended processing times.
Organic solvent extraction	Simple, affordable, and moderately effective; suitable for small-scale operations.	Slow; requires a lot of solvent; prevents agitation from speeding up the process; uses organic solvents, which might have harmful or unexpected consequences on the end product.
Cold pressing	Easy and low-cost, it may be used for making citrus oils.	Failure to fully extract oil; impractical for low-oil samples.
Supercritical fluid extraction	Extraction without the use of harmful solvents takes less time and does not need their use.	Exorbitant prices for machinery, set-up, and servicing.
Microwave-assisted extraction	The extraction yield increased, and the process is easily repeatable, manipulatable, needs little solvent, requires little energy, and is energy-efficient.	At the end, it will need to filter or centrifuge the mixture.
Ultrasound-assisted extraction	Quicker extraction, less solvent used, better mass transfer, deeper solvent penetration, and lower costs (in comparison to SCFE and MAE) all characterize this easy and cheap method.	Low performance, high energy efficiency, and poor scalability.

Table S2. Statistical design of split plot for studying the essential oil extracted from Eucalyptus hybrids grown at each of the campus of King Abdulaziz University (KAU), Hada Al-Sham, and Briman by using two extraction methods, namely, microwave-assisted steam extraction (MASE) and electric-steam extraction (ESE) as repeated for three blocks.

**Block 1**		**Block 2**		**Block 3**	
Main plot	Sub-plot (h)	Main plot	Sub-plot (h)	Main plot	Sub-plot (h)
KAU	MASE	Hada Al-Sham	MASE	Briman	ESE
	ESE		ESE		MASE
Hada Al-Sham	ESE	Briman	ESE	KAU	MASE
	MASE		MASE		ESE
Briman	ESE	KAU	ESE	Hada Al-Sham	MASE
	MASE		MASE		ESE

Table S3. Statistical design of split-split plot for the encapsulation investigation to study the difference between two polymeric encapsules, namely, guar gum-based encapsules (GGBEs) and alginate-based encapsules (ABEs), which were uploaded with different concentrations of 1,8-cineol in a concentration of of 50, 100, 150, and 200 μL for a duration of 1, 1.5, and 2h as repeated for three blocks.

Block 1			Block 2			Block 3		
Main plot	Sub-plot (h)	Sub-sub-plot (μL)	Main plot	Sub-plot (h)	Sub-sub-plot (μL)	Main plot	Sub-plot (h)	Sub-sub-plot (μL)
GGBE	1h	50	ABE	2h	100	GGBE	1.5h	200
		100			200			100
		150			150			50
		200			100			1500
	1.5h	50		1h	50		2h	50
		100			200			100
		150			150			150
		200			100			200
	2h	50		1.5h	200		1h	50
		100			100			100
		150			150			150
		200			50			200
ABE	1h	50	GGBE	2h	100	ABE	1.5h	150
		100			200			100

		150			50			50
		200			200			200
	1.5h	50		1.5h	50		1h	50
		100			200			150
		150			150			200
		200			100			100
	2h	50		1h	50		2h	150
		100			150			100
		150			100			50
		200			200			200

Figure S1. The electric components of the microwave generator unit (MGU) used for heating the

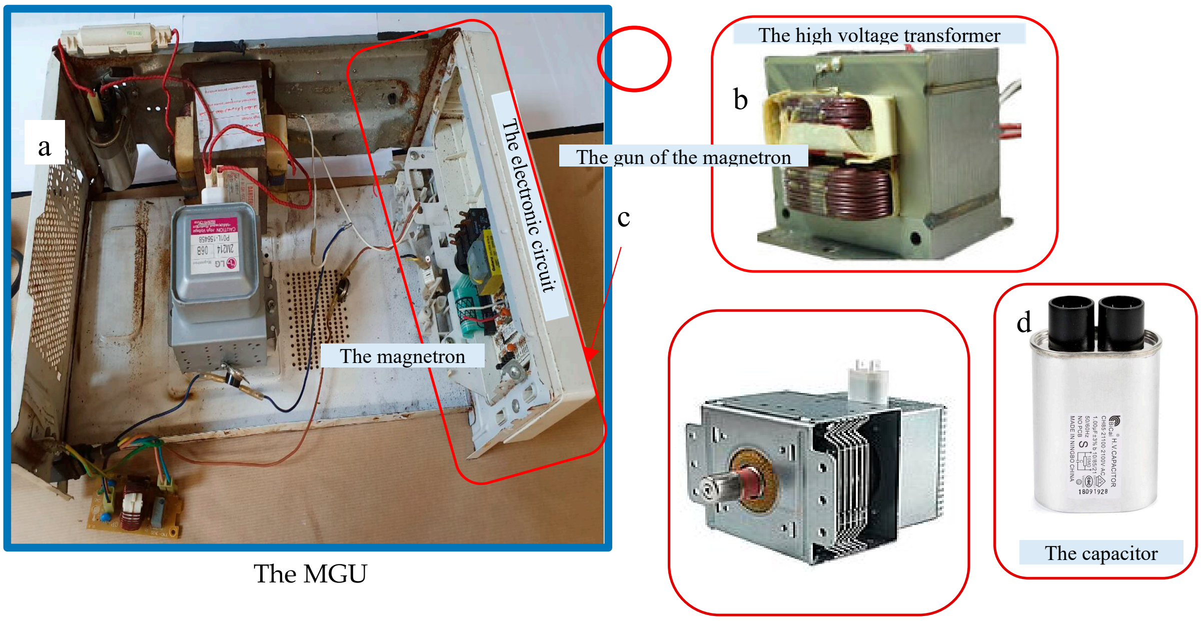

extraction vessel of the MASD: a) overall image of the MGU, b) the high-voltage magnetron, c) the high-voltage transformer, and d) the high-voltage capacitor.

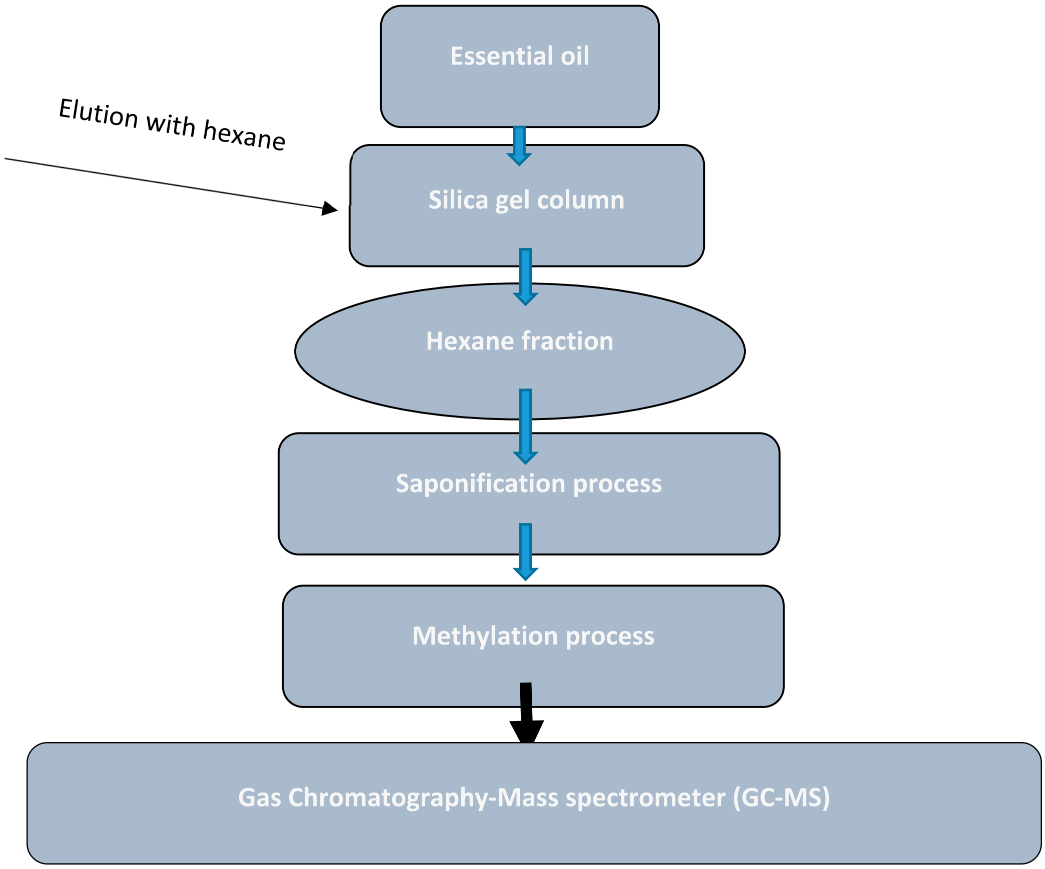

Figure S2. Preparation of methyl esters of the essential oils for analysis by GC-MS.
